# The Use of Artificial Intelligence in the Differentiation of Malignant and Benign Lung Nodules on Computed Tomograms Proven by Surgical Pathology

**DOI:** 10.3390/cancers12082211

**Published:** 2020-08-07

**Authors:** Yung-Liang Wan, Patricia Wanping Wu, Pei-Ching Huang, Pei-Kwei Tsay, Kuang-Tse Pan, Nguyen Ngoc Trang, Wen-Yu Chuang, Ching-Yang Wu, ShihChung Benedict Lo

**Affiliations:** 1 Department of Medical Imaging and Intervention, Linkou Chang Gung Memorial Hospital, College of Medicine, Chang Gung University, Taoyuan 333, Taiwan; pww@cgmh.org.tw (P.W.W.); spookie@cgmh.org.tw (P.-C.H.); pan0803@cgmh.org.tw (K.-T.P.); 2 Department of Public Health and Center of Biostatistics, College of Medicine, Chang Gung University, Taoyuan 333, Taiwan; tsay@mail.cgu.edu.tw; 3 International Ph.D. Program in Medicine, College of Medicine, Taipei Medical University, 250 Wu-Hsing Street, Taipei 110, Taiwan; drnguyenngoctrang@gmail.com; 4 Department of Anatomic Pathology, Linkou Chang Gung Memorial Hospital, College of Medicine, Chang Gung University, Taoyuan 333, Taiwan; s12126@cgmh.org.tw; 5 Division of Thoracic & Cardiovascular Surgery, Linkou Chang Gung Memorial Hospital, College of Medicine, Chang Gung University, Taoyuan 333, Taiwan; wu.chingyang@gmail.com; 6 Arlington Innovation Center: Health Research, Virginia Polytechnic Institute and State University, Arlington, VA 22203, USA; dcben0@gmail.com

**Keywords:** computer-aided detection and analyzer, artificial intelligence, CT, lung cancer screening, vessel suppression

## Abstract

The purpose of this work was to evaluate the performance of an existing commercially available artificial intelligence (AI) software system in differentiating malignant and benign lung nodules. The AI tool consisted of a vessel-suppression function and a deep-learning-based computer-aided-detection (VS-CAD) analyzer. Fifty patients (32 females, mean age 52 years) with 75 lung nodules (47 malignant and 28 benign) underwent low-dose computed tomography (LDCT) followed by surgical excision and the pathological analysis of their 75 nodules within a 3 month time frame. All 50 cases were then processed by the AI software to generate corresponding VS images and CAD outcomes. All 75 pathologically proven lung nodules were well delineated by vessel-suppressed images. Three (6.4%) of the 47 lung cancer cases, and 11 (39.3%) of the 28 benign nodules were ignored and not detected by the AI without showing a CAD analysis summary. The AI system/radiologists produced a sensitivity and specificity (shown in %) of 93.6/89.4 and 39.3/82.1 in distinguishing malignant from benign nodules, respectively. AI sensitivity was higher than that of radiologists, though not statistically significant (*p* = 0.712). Specificity obtained by the radiologists was significantly higher than that of the VS-CAD AI (*p* = 0.003). There was no significant difference between the malignant and benign lesions with respect to age, gender, pure ground-glass pattern, the diameter and location of the nodules, or nodules <6 vs. ≥6 mm. However, more part-solid nodules were proven to be malignant than benign (90.9% vs. 9.1%), and more solid nodules were proven to be benign than malignant (86.7% vs. 13.3%) with statistical significance (*p* = 0.001 and <0.001, respectively). A larger cohort and prospective study are required to validate the AI performance.

## 1. Introduction

Lung cancer is currently the leading global cause of cancer-related death [1,2]. Current five-year survival estimates for non-small cell lung cancer range from 73% for Stage IA disease to 13% for Stage IV disease [3]. In the earliest stages, the lung cancer can manifest as a pure ground-glass nodule (GGN), part-solid, or solid nodule [4,5]. The tumor’s solid component is more strongly correlated with invasive pathologic characteristics and associated with the worse survival rates [4,5,6,7,8]. The identification of patients with lung cancer in early stages or Stage IA is associated with better prognosis for disease-free survival [9,10]. International Early Lung Cancer Action Program Investigators reported that the early detection and surgical excision of lung cancer presenting as GGN or part-solid nodules can provide a lung cancer-specific survival rate of up to 100% [11,12]. The early detection of lung cancer in patients using low-dose computed tomography (LDCT) was shown to be highly effective according to the report of National Lung Screening Trial (NLST) in 2011, with a relative reduction in lung cancer mortality by 20% compared to chest radiography [13]. Since then, the United States Preventive Services Task Force has recommended annual screening for lung cancer in asymptomatic adults aged 55 to 80 years who have a 30-pack/year smoking history and currently smoke or had quit within the past 15 years [14].

LDCT has the benefit of depicting pulmonary structures without superimposition while requiring less radiation than conventional CT [15]. However, pulmonary nodules may still be overlooked due to various factors. Aside from observer performance, nodule characteristics such as small dimensions, poor conspicuity, ill-defined margins, and central locations adjacent to vessels may all lead to missing lung cancers on CT [16]. Particularly, large sets of images taken by LDCT were associated with visual and mental fatigue for radiologists that may cause intellectual or interpretative errors during routine clinical practice [17]. Missing a lung nodule/cancer in a radiological examination is highly concerning and remains one of the most common causes of malpractice claims against radiologists [18,19].

Research in computer-aided detection (CAD) has been carried out for more than two decades to improve the efficacy and efficiency of detecting small lung nodules [20]. Advanced detection algorithms and high-speed calculations have allowed for the development of new CAD systems to be much more powerful, and gave them the potential to aid radiologists in the diagnosis of lung nodules [20,21]. The use of CAD alone is not generally accepted in clinical practice [22]. However, the vessel-suppressed (VS)-CAD system (i.e., ClearRead CT software) that was approved by the US Food and Drug Administration (FDA) in 2016 takes a different approach, by suppressing normal structures within the lungs, which are mostly vascular. This system seeks to improve the detectability of small lung nodules. If artificial intelligence (AI) or CAD systems could accurately pinpoint lung nodules on CT, this technology would not only assist radiologist performance in nodule detection, but also reduce their clinical reading time [22,23]. Assuming that deep-learning AI was trained to only focus on lung nodule analysis and was capable of distinguishing malignant from benign nodules, the aims of this project were to (1) investigate and compare the performance of the VS-CAD AI with radiologists in differentiating malignant from benign lung lesions, and (2) analyze the sensitivity and specificity of VS-CAD AI in detecting malignancies that were excluded from its analysis summary. To the best of our knowledge, this is the first study attempting to assess the VS-CAD AI’s ability to stratify and detect lung nodules that were all proven by surgical pathologies.

## 2. Results

### 2.1. Baseline Description: 75 Nodules with Final Surgical Pathology

All 75 nodules in the 50 cases, comprised of 47 malignant nodules and 28 benign lesions, which were excised and proven by surgical pathology, were well highlighted on VS images. The 47 malignancies were made up of 23 adenocarcinomas, 18 minimally invasive adenocarcinomas (MIAs), and six adenocarcinomas in situ (AISs). The 28 benign lesions included 10 inflammations, seven intrapulmonary nodes (IPNs), six fibrosis, and one case each of atypical adenomatous hyperplasia (AAH), primary benign leiomyoma, primary benign clear-cell tumor, atelectasis, and granuloma.

Of the 50 subjects (35 malignant cases) with 75 nodules, there was no statistically significant difference between the benign and malignant cases or nodules with respect to age (57.47 ± 10.97 vs. 55.06 ± 8.33, *p* = 0.399), gender (*p* = 0.754), nodule diameter (7.16 ± 3.29 vs. 7.82 ± 3.06, *p* = 0.193), pure ground-glass patterns (34.2% vs. 65.8%, *p* = 0.743), upper-lobe vs. non-upper lobe location (*p* = 0.812), or peripheral vs. central location (*p* = 0.772) (Table 1). Sixty-one nodules summarized by the AI analyzer were reviewed and showed that there was no significant difference in the 61 nodule sizes measured manually or by the AI analyzer (7.83 ± 3.06 vs. 8.13 ± 3.49, *p* = 0.624) with a Pearson correlation coefficient of 0.926.

Among the 75 nodules, more part-solid nodules were proven to be malignant than benign (90.9% vs. 9.1%), and more solid nodules were proven to be benign than malignant (86.7% vs. 13.3%) with statistical significance in multivariate analysis using logistic regression (*p* = 0.001 and <0.001, respectively) (Table 1). In 2017, the Fleischner Society suggested that a nodule <6 mm in low-risk adults >35 years old generally needs no further follow-up [24,25]. These guidelines also suggest that a small lung nodule in females (or in the upper lobe) has a high risk of malignancy, and requires close observation or a follow-up CT scan. However, our original clinical-study cases were performed during the period of November 2014 to August 2018. In our cohort, there was no significant difference in the prevalence of malignancy between nodules <6 mm and those ≥6 mm (57.7% vs. 65.3%, *p* = 0.618).

### 2.2. Proposed Application of VS-CAD AI System in Detecting Lung Cancer and Excluding Benign Lesions

**Figure 1:**
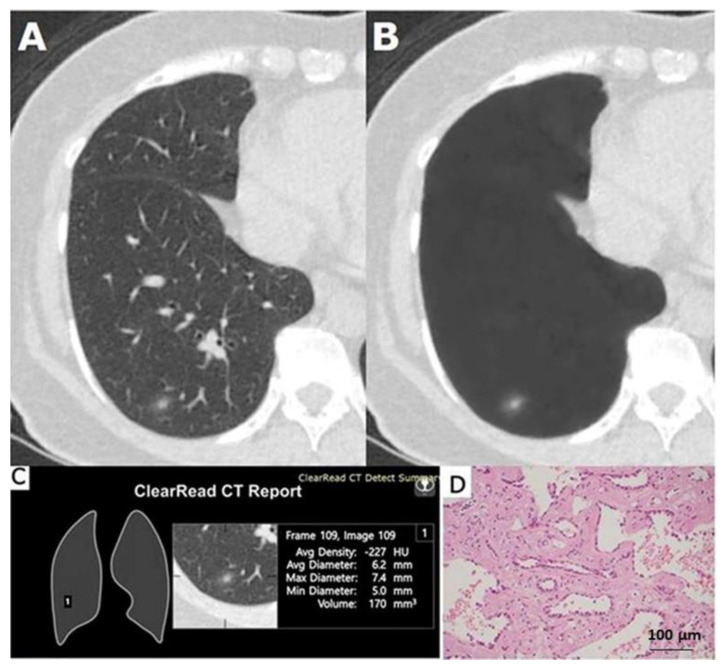
A 59-year-old female was found to have a 6.2 mm part-solid nodule in the right lower lobe (RLL) which was proven to be an acinar predominant adenocarcinoma. (**A**) Low-dose computed tomogram. (**B**) Its vessel-suppressed image showed a 6.2 mm part-solid nodule left in RLL. (**C**) The vessel-suppressed and computer-aided detection artificial intelligence analyzer indicated that the nodule was actionable. (**D**) Microphotograph of the specimen showed acinar predominant adenocarcinoma (hematoxylin and eosin stain, ×200).

**Figure 2:**
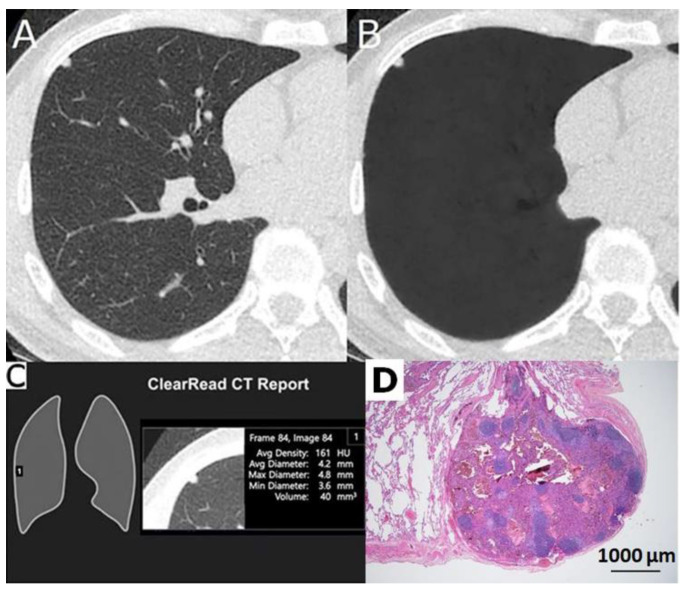
A 44-year-old male with a 4.2 mm well defined solid nodule with smooth outline due to intrapulmonary node in right middle lobe (RML). (**A**) Low-dose computed tomogram and (**B**) its vessel-suppressed image showed a 4.2 mm solid sub-pleural nodule in RML. (**C**) Vessel-suppressed and computer-aided detection artificial intelligence system alerted it as an actionable nodule. (**D**) Microphotograph of the specimen showed an intrapulmonary node (hematoxylin and eosin stain, ×20).

**Figure 3:**
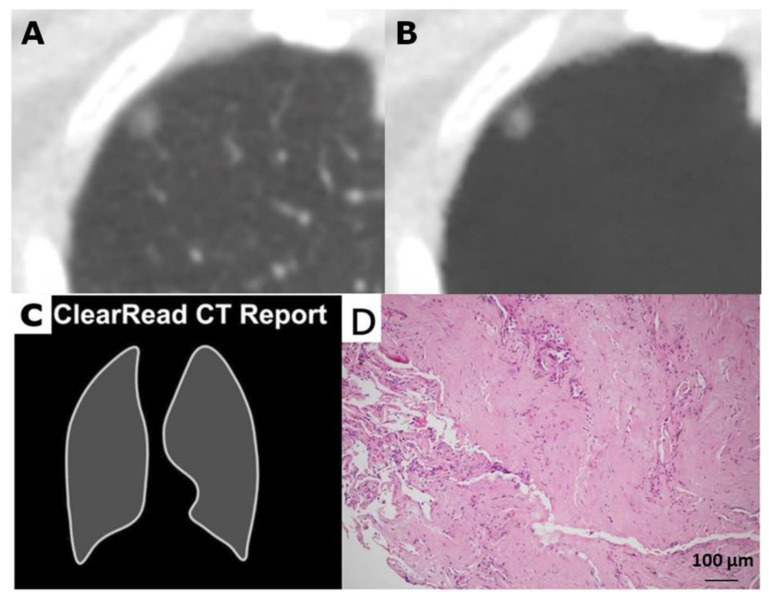
A 57-year-old female with a true negative cancer result ignored by the vessel-suppressed and computer-aided detection (VS-CAD) artificial intelligence (AI) for a small nodule in right upper lobe (RUL). (**A**) Low-dose computed tomogram and (**B**) the vessel-suppressed image revealed a 7 mm well defined and smooth-outlined ground-glass nodule in RUL. (**C**) The VS-CAD AI analyzer treated it as a non-actionable nodule and did not prompt an analysis summary. (**D**) The nodule was proved to be a benign lesion with inflammation, fibrosis and anthracosis by surgical pathology (hematoxylin and eosin stain, ×100).

**Figure 4:**
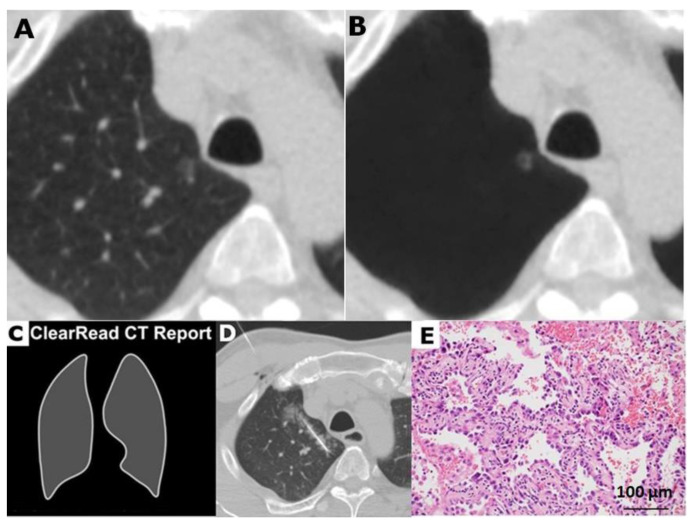
A 58-year-old male with minimally invasive adenocarcinoma in right upper lobe (RUL). (**A**) Low-dose computed tomogram and (**B**) its vessel-suppressed image showed a 6.2 mm ground-glass nodule in RUL. The nodule is relatively ill defined and suspiciously lobulated. (**C**) The vessel-suppressed and computer-aided detection artificial intelligence analyzer determined it as a non-actionable nodule and did not prompt an analysis summary. (**D**) Computed tomography guided needle localization was performed. (**E**) Microphotograph of the specimen showed minimally invasive adenocarcinoma (hematoxylin and eosin stain, ×200).

Of the 75 surgically excised nodules, 61 nodules (81.3%) were marked as positive (Figure 1 and Figure 2), and 14 nodules (18.7%) were unmarked in each analysis summary of the AI system (Figure 3 and Figure 4). Among these 14 negative or nonactionable nodules, 11 were benign and three were malignant (one adenocarcinoma, one MIA, and one AIS measuring 5.7, 6.4, and 6.8 mm in diameter, respectively). All three malignant nodules were GGNs, and were well delineated on the LDCT and VS images (Figure 4). The VS-CAD AI analyzer that produced the analysis summary ignored three (6.4%) of the 47 malignant nodules (Figure 4) and 11 (39.3%) of the 28 benign lesions (Figure 3) with statistically significant difference (*p* = 0.001). Of those 11 benign nodules, seven were GGNs, two were solid, and two were part-solid. The definition of “false-positive” in this study is different from that typically used in the aforementioned nodule-detection studies. Any benign lesion marked as an actionable nodule by the AI analyzer was treated as a false-positive classification in this work.

Table 2 shows the results of the VS-CAD AI and radiologists in differentiating malignant from benign nodules. When using the VS-CAD AI system as a classifier to distinguish malignant from benign nodules, the system produced a sensitivity of 93.6% for cancer detection, specificity of 39.3%, a positive predictive value of 72.1%, a negative predictive value of 78.6%, and an accuracy of 73.3%. The corresponding figures performed by radiologists in detecting lung cancers were 89.4%, 82.1%, 89.4%, 82.1%, and 86.7%, respectively. The sensitivity of the VS-CAD analyzer was higher than that of radiologists, but not statistically significant (*p* = 0.712). Specificity obtained by radiologists were significantly higher than that of the VS-CAD AI (*p* = 0.003).

Twenty-six (11 benign and 15 malignant) of the 75 nodules were <6 mm. Of these 26 lesions which were <6 mm, 11 benign lesions (42.3%) included five IPNs that were solid, three fibrosis, and three inflammatory processes that featured ground-glass opacities; six of the 11 benign lesions were not marked by the VS-CAD AI analyzer. The 15 lung cancers (57.7%) comprised seven MIAs, six adenocarcinomas, and two AISs, and all but one was marked by the VS-CAD AI analyzer. Of the 15 lung cancers, six were part-solid and nine featured ground-glass opacities.

### 2.3. Synchronous Multiple Primary Lung Cancers (SMPLCs)

Out of the 50 patients, nine (18%) of them (aged 43 to 60, mean 54.6; seven females and two males) had a total of 21 SMPLCs (12 adenocarcinomas, six MIAs, and three AISs). Among these, 13 were GGNs, seven were part-solid nodules and one was solid. Of the nine patients, six had two SMPLCs each, and three had three SMPLCs each. The mean size of these nodules was 7.00 ± 2.35 mm.

## 3. Discussion

In this study, all pathologically proven lung nodules were well delineated on the VS images. Only two (2.7%) of the 75 nodules were not detected in the initial interpretation of the LDCT images. Lung cancer on CT images can be missed due to detection or interpretation errors. Failure to identify a nodule is considered a detection error, whereas dismissing a potentially malignant nodule as a benign finding that needs no further evaluation is considered an interpretation error. Interpretation errors usually occur when the morphologic structure of the abnormality is similar in appearance to that of a benign lesion, such as a scar, fibrosis, or cyst [20]. CAD systems are classified into two groups, computer-aided-detection (CADe) and computer-aided-diagnosis (CADx) systems [20]. In order to obtain a systematically high-performance result from a CAD system, it is technically essential to apply a prescreen function to and minimize destruction in computer analyses [26,27]. The first component of the VS-CAD AI in this study was a specialized 3D convolutional-neural-network system that predicted normal lung vascular structure, and the second component was a modular neural-network system inherently possessing a feature-extraction mechanism, which classified regions on the basis of their attenuation and other features. These regions were then measured for characterization [28]. Other CADx systems directly extract specific predefined characteristics from the images and use a classifier to estimate the probability of malignancy [29]. Several investigators reported a wide range of sensitivities using CAD, from 73% to 96.7%, with false-positive (FP) detection rates of 0.55 to 8.2 per scan for detecting lung nodules [30,31,32,33]. The sensitivity of the nodule detection was 78.1% for a double reading and 96.7% for CAD. A total of 69.7% of nodules undetected by readers were attached nodules, among which 78.3% were vessel attached [30]. This suggests the significance of vessel suppression, which is the goal of the first component of the AI system used in this study.

This study was motivated by the present system’s well-known analysis capabilities, with it being the first FDA-approved system for the detection of all nodule types. Since most cancer opacities inherently possess more features than benign opacities, the AI might have learned those cancer features, thereby leading to a higher sensitivity in cancer detection. The results of this study showed that the VS-CAD AI system may have potential for the analysis of malignant and benign lung nodules on LDCT, although this capability deviates from its current intended use.

The sensitivity of the VS-CAD AI analyzer in detecting lung cancer or malignancies in this study was 93.6%, which is slightly higher than the 89.5% reported in another study using an early version of VS-CAD AI software [22]. In this study, the performance of radiologists in differentiating malignant from benign nodules might have been biased and partially affected by the following scenarios: first, hematogenous metastases that usually feature well defined solid nodules were excluded from this study. Second, part-solid nodules have a high likelihood of malignancy [24], reported to be 78.1% to 93.3% [34,35]. In our dataset, 90.1% of part-solid nodules were cancers. In addition, the use of the VS-CAD AI system had the advantage of a 26% reduction in reading time and a 29% reduction of the missing nodules in a lung cancer-screening study setting, though there was a false-positive detection rate of 0.58 per scan (Table 3) [22].

The clinical efficacy and reliability of many CAD systems for the detection of lung nodules were previously assessed. Jacobs et al. reported a sensitivity of 82% in detecting lung nodules by a CAD system on 888 thoracic CT scans [38]. The study by Li et al. showed that a deep-learning-based CAD system without vessel suppression increased the sensitivity of nodule detection from 77.5% to 84.3% for nodules <5 mm, and from 88% to 96.5% for nodules ≥5 mm [36]. This deep-learning-based CAD system also showed a higher detection rate than double reading regardless of the nodule size (86.2% vs. 79.2%; *p* < 0.001) [36]. The false-positive nodule detection rates were in the range of 1.53 to 4 per scan for several tested CAD systems [36,37,38]. In contrast, the VS-CAD AI system produced a very low false-positive rate of 0.58 per scan [22]. Table 3 shows the parameters and summarized performance results of several currently commercially available AI systems for the detection of lung nodules.

Among the 75 nodules, more part-solid nodules were proven to be malignant than benign (90.9% vs. 9.1%), with statistical significance in multivariate analysis using logistic regression (*p* = 0.001), a finding that was consistent with previous reports [7,8,34,35]. Since vessels involved in a part-solid nodule could be effectively traced and suppressed by the VS-CAD AI system, more visible true nodule characteristics result in a more accurate interpretation in clinical practice. This result also indirectly reflects our assumption that the VS-CAD AI analyzer was trained to be more sensitive to opacities with rich features, such as part-solid nodules possessing low- and high-frequency component textures. This result could allow for the current system to supplant several other unsuccessful AI systems, as a substantial number of their missed nodules (30%) were part-solid, as reported by Jacobs et al. [38].

There are several limitations to our study. First, this study was based on a single center, and the sample size was rather small. A larger cohort and prospective study are required to validate our results. Second, the VS-CAD AI system was employed to evaluate 50 pathologically proven LDCT cases as a laboratory study that does not represent regular clinical practice. Third, the AI module was not tested in a variety of different patient populations. Lastly, the results and conclusions obtained in this study were limited to an AI module provided by one vendor.

## 4. Materials and Methods

### 4.1. Study Population and Data Collection

This retrospective study was approved by the institutional review board (ethical code is: 201801886B0D001). During the period from November 2014 to August 2018, a total of 561 patients that underwent CT-guided localization of their lung nodules had subsequent video-assisted thoracic surgery and pathological examination. The indications of chest CT or causes of nodules detected in the lungs of those 561 patients included LDCT health examination at one’s own expense (n = 207), malignant neoplasms of other organs (n = 127), chief complaints other than respiratory symptoms (n = 103), symptoms or signs of respiratory diseases (n = 68), and follow-up CT of lung cancer after treatment (n = 56). For data collection in this study, exclusion criteria were any one of the following: subjects having (1) a previous history of thoracic surgery and/or (2) lung nodules >2 cm in maximal diameter or a final pathological diagnosis with metastases (or inconclusive). Ultimately, 50 patients with 75 lung nodules ≤2 cm met all the following inclusion criteria: (1) the LDCT was available within 3 months before surgical excision of the lung nodule; (2) the LDCT was performed from the lung apex to the lung base with a slice thickness and intervals of ≤2.5 mm; and (3) each lung nodule was pathologically proven. Of the 50 patients, 18 were males, and ages ranged from 37 to 80 (mean 55.8) years. Any patient having a malignant nodule was designated as a malignant case.

### 4.2. CT Techniques and Image Acquisition

All CT images were obtained using a helical (1) Bright Speed 16-row CT scanner (GE Medical Systems, Milwaukee, Wisconsin) at Linkou Chang Gung Memorial Hospital with detector width of 0.625 mm, (2) a 320-row detector CT scanner (Aquilion ONE, Toshiba Medical Systems, Otawara, Japan) at Linkou and Taoyuan Chang Gung Memorial Hospitals, or (3) a 64-detector CT scanner (Aquilion CXL 128 slices, Toshiba Medical Systems, Otawara, Japan) at Taipei Chang Gung Memorial Hospital. Both the Aquilion ONE and Aquilion CXL systems had a gantry-rotation time of 0.35 s, and each detector’s width was 0.5 mm. CT images were obtained with the scanning parameters for LDCT suggested by the American College of Radiology and the Society of Thoracic Radiology [39]: 120 kVp, 20–40 mAs, pitch 0.8 to 1.5, field of view (FOV) from the lung apex to the costophrenic angle in lateral scout view, and an image-slice thickness ≤2.5 mm without a gap, a soft or intermediate reconstruction kernel, and transverse images displayed in the lung window (1500/−500) and mediastinal window (500/50). The mean radiation dose or CT dose index volume (CTDIvol) of each scan was 2.19 ± 0.66 mGy, which complied with the guideline of less than 3 mGy for standard-sized subjects weighing up to 160 lb and 5 feet 7 inches in height. The locations of the lung nodules were identified, assessed, and reported if present in a lung window by the radiologist.

### 4.3. Retrospective Study with Laboratory Setting

The VS-CAD AI system was trained with a large number of nodules as for any lung nodule AI system. Its training also included lung cancer regions and their digitally transformed versions, spanning a large range of size, attenuation, and texture. The second component of the VS-CAD AI system was a modular system of neural networks inherently possessing a feature-mapping analyzer that focused on stratifying nodules without the interference of vessels [28].

All 50 CT cases were processed by the VS-CAD AI system to generate 50 sets of results. Each set in the study result consisted of (1) another set of the same CT images whose vessels were suppressed and (2) a tabulated analysis summary if a cancer/nodule was detected. These two parts of the results were produced by two separate functions of the VS-CAD AI system, as described below.

#### 4.3.1. Vessel-Suppressed CT as VS-CAD AI Postprocessing

The first part of the VS-CAD AI system was developed to suppress 3D vessel structures in thoracic CT. Through a combination of AI and nodule simulation, the VS-CAD AI system was capable of separating vascular structures from lung parenchyma. This way, lung abnormalities left on vessel-suppressed CT images are much easier to identify by radiologists and computer-detection processes such as the CAD analyzer provided by the VS-CAD AI system.

#### 4.3.2. Detection and Output of CAD AI Analyzer

When the second part of the AI system determined that a region of interest (ROI) contained a nodule, it created a mark with an attached analysis summary to both the original and vessel-suppressed CT series. Specifically, the marked ROI, as well as its average density, diameter, and volume, are shown on the system’s tabulated summary as an actionable nodule (Figure 1 and Figure 2). This is stated in its intended use (as opposed to a nonactionable ROI; Figure 3 and Figure 4). A maximal total of 20 nodules (5 lesions on a page) could be prompted by the analysis summary of the VS-CAD AI system.

### 4.4. Lung-Nodule Characteristics and Images Interpretation

**Figure 5:**
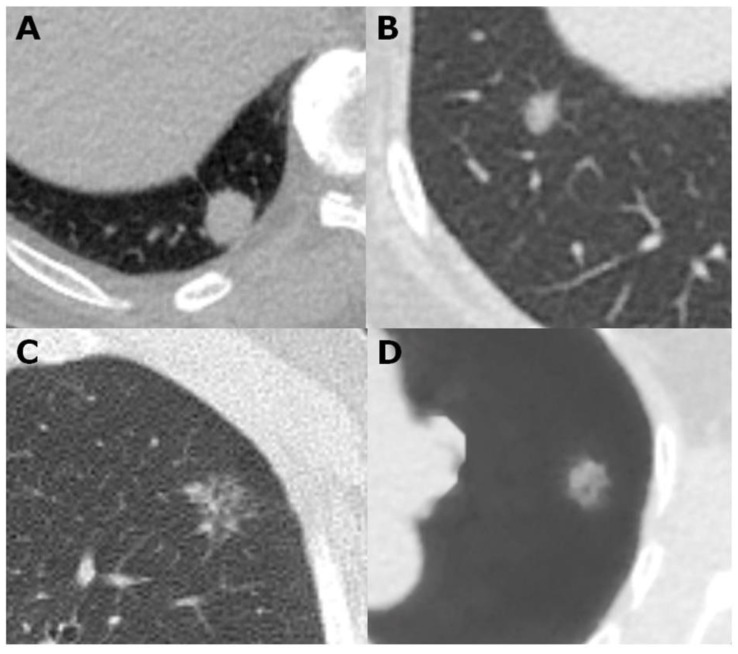
(**A**) A 1.8 cm invasive adenocarcinoma in the right lower lobe featuring a solid nodule with speculation. (**B**) An indistinct 8.2 mm part-solid nodule with speculation in the right lower lobe due to adenocarcinoma in situ. (**C**) A 1.8 cm speculated ground glass nodule in the left upper lobe due to lepidic predominant adenocarcinoma. (**D**) A vessel-suppressed image shows a 1.3 cm ground glass nodule due to lepidic predominant adenocarcinoma in the left upper lobe which is characterized by indistinct margin, lobulation and speculation.

The average diameter of each nodule (measured manually and by the AI analyzer) was recorded. The location of each nodule was categorized as either peripheral (located in the pulmonary parenchyma within 2 cm from the pleura) or central. Nodules were classified as solid (Figure 2 and Figure 5A), part-solid (Figure 1 and Figure 5B), or ground-glass (Figure 3, Figure 4 and Figure 5C,D). Nodules on LDCT images were classified as either malignant or benign by the two radiologists (having 38 and 25 years of experience in thoracic-image interpretation) in consensus, who were blinded to the pathological results. Since metastatic nodules were excluded from this study, the solid, round, or triangular nodules with a well-defined and smooth outline were tentatively designated as benign (Figure 2), and solid nodules with a lobulated or speculated margin were classified as malignant (Figure 5B) [40]. A part-solid nodule was classified as malignant [24,34,35,40,41] (Figure 1 and Figure 5B) unless it was associated with pneumonitis. A GGN was classified as malignant when it was associated with any or the combination of the following features: lobulation, speculation, spine-like process, margin with coarse interface, plural indentation, vascular convergence, bubbly appearance, or air-containing space (Figure 4 and Figure 5C,D) [40,41,42].

### 4.5. Statistical Analysis

All the continuous variables were expressed as the means ± standard deviation and compared using an independent two-sample t-test. Categorical variables were expressed as numbers and percentages, and were analyzed by a chi-squared or Fisher’s exact test when appropriate. A Pearson correlation coefficient test was performed to analyze the relationship between the nodule diameters obtained by the manual measurements and by the AI. Univariate and multivariate analyses by logistic regression were performed to analyze nodule types, malignant or benign.

The malignant and benign nodules were designated as positive and negative results, respectively. The corresponding results of the AI in differentiating malignant from benign lesions were assessed (although deviating from the VS-ACD, AI’s current intended use) and tabulated as true-positive, true-negative, false-positive, or false-negative. Sensitivity, specificity, positive predictive values, negative predictive values, and accuracy were calculated. A *p* value of <0.05 was considered statistically significant for all evaluations. Statistical analysis was performed with SPSS 20.0 software for Windows (SPSS, Inc., Chicago, IL, USA).

## 5. Conclusions

The first component of the VS-CAD AI system delineated all pathologically proven lung nodules. The second component identified pathologically proven cancers with high sensitivity. In the initial interpretation of the LDCT images, very few pathologically proven nodules were not detected by radiologists. The study showed that the VS-CAD AI seems to have a high sensitivity in identifying malignant lung nodules and it also characterizes quite a few benign nodules. Furthermore, there was no significant difference in the sensitivity between the VS-CAD AI system and radiologists. However, the radiologists had a significantly better performance than that of the VS-CAD AI in specificity. A larger cohort and prospective study will be required to validate the performance of the vessel suppression-based AI analyzer for lung cancer detection.

## Figures and Tables

**Table 1:** The demographic data of 50 patients and imaging findings of 75 nodules shown on low-dose computed tomograms as well as their vessel-suppressed images.

Variables	Benign	Malignant	*p*-Value
Patients (*n* = 50)			
Age (mean ± SD)	57.47 ± 10.97	55.06 ± 8.33	0.399
Gender (*n* = 50)	15 (30%)	35 (70%)	
Female (*n* = 32)	9 (28.1%)	23 (71.9%)	0.754
Male (*n* = 18)	6 (33.3%)	12 (66.7%)	
Nodules (*n* = 75)	28 (37.3%)	47 (62.7%)	
Diameter (mm) (mean ± SD)	7.16 ± 3.29	7.82 ± 3.06	0.193
≥6 mm	17 (34.7%)	32 (65.3%)	0.618
<6 mm	11 (42.3%)	15 (57.7%)	
Ground glass nodule (*n* = 38)	13 (34.2%)	25 (65.8%)	0.743
Peripheral (*n* = 31)	11 (35.5%)	20 (64.5%)	1.000
Central (*n* = 7)	2 (28.6%)	5 (71.4%)	
Part-solid nodule (*n* = 22)	2 (9.1%)	20 (90.9%)	0.001
Peripheral (*n* = 15)	1 (6.7%)	14 (93.3%)	1.000
Central (*n* = 7)	1 (14.3%)	6 (85.7%)	
Solid (*n* = 15)	13 (86.7%)	2 (13.3%)	<0.001
Peripheral (*n* = 13)	11 (84.6%)	2 (15.4%)	1.000
Central (*n* = 2)	2 (100%)	0 (0%)	
Nodules location			
Upper lobe (*n* = 33)	13 (39.4%)	20 (60.6%)	0.812
Not upper lobe (*n* = 42)	15 (35.7%)	27 (64.3%)	
Peripheral (*n* = 59)	23 (39.0%)	36 (61.0%)	0.772
Central (*n* = 16)	5 (31.2%)	11 (68.8%)	

**Table 2:** The results and comparison of the vessel-suppressed computer-aided detection artificial intelligence (VS-CAD AI) system and radiologists in differentiating malignant from benign nodules.

**AI vs. Radiologists**	**Malignant vs. Benign**		**Pathology**		**Total**
			Malignant	Benign	
			47	28	75
VS-CAD AI	Malignant		44	17	61
	Benign		3	11	14
Radiologists	Malignant		42	5	47
	Benign		5	23	28
**AI vs. Radiologists**	**Sensitivity**	**Specificity**	**Positive** **Predictive** **Value**	**Negative** **Predictive Value**	**Accuracy**
VS-CAD AI	93.6% (82.5–98.7%)	39.3% (21.5–59.4%)	72.1% (65.6–77.9%)	78.6% (52.8–92.3%)	73.3% (61.9–82.9%)
Radiologists	89.4% (76.1–96.0%)	82.1% (62.4–93.2%)	89.4% (76.1–96.0%)	82.1% (62.4–93.2%)	86.7% (76.4–93.1%)
*p* value	0.712	0.003	0.050	0.999	0.066

Percentages in parenthesis indicate proportions with 95% confidence interval.

**Table 3:** Parameters and performance of three commercially available artificial intelligence (AI) systems for the detection of lung nodules on thoracic CT.

AI Software for Screening Lung Cancer	Study Data Source	# of Cases (with nodule)	# of Non- nodule Cases	AI Sensitivity	AI FP Rate (per scan)	Evaluated Nodule Size	Radiologists Baseline Performance	Remarks and References #
VS-CAD AI	Selected cases from the NLST CT arm	108 (179)	206	90% (cancer) 82% (nodule)	0.58	≥0.5 mm	60.1% with 0.17 FP/scan	Vessel suppression prior to nodule detection and analysis [22]
σ-Discover	Smokers in Beijing and Shenzhen met the inclusion criteria like the NLST	314 (812)	32	86.2%–96.5% (nodule)	1.53	≥0.3 mm and ≥0.5 mm	79.2–88% with 0.13 FP/scan	Vessel and artifacts were two main causes of FPs in both σ-Discover and after double reading [36]
Syngo Lung CAD Manager	Early Detection Research Network – NYU study cohort	39 (134)	4	67%–66% (nodule)	2.8	≥0.3 mm and ≥0.5 mm	44–48% with 0.07 FP/scan	Majority of FPs accepted by radiologists were vessels and peribronchial findings [37]

FP = false-positive. VS-CAD AI = vessel-suppressed computer-aided detection artificial intelligence. # = Number. NLST = National Lung Screening Trial. NYU = New York University.
